# An efficient scRNA-seq dropout imputation method using graph attention network

**DOI:** 10.1186/s12859-021-04493-x

**Published:** 2021-12-07

**Authors:** Chenyang Xu, Lei Cai, Jingyang Gao

**Affiliations:** College of Information Science and Technology, Beijing University of Chemical Technology, Beijing, People’s Republic of China

**Keywords:** scRNA-seq, Dropout imputation, Graph attention convolution

## Abstract

**Background:**

Single-cell sequencing technology can address the amount of single-cell library data at the same time and display the heterogeneity of different cells. However, analyzing single-cell data is a computationally challenging problem. Because there are low counts in the gene expression region, it has a high chance of recognizing the non-zero entity as zero, which are called dropout events. At present, the mainstream dropout imputation methods cannot effectively recover the true expression of cells from dropout noise such as DCA, MAGIC, scVI, scImpute and SAVER.

**Results:**

In this paper, we propose an autoencoder structure network, named GNNImpute. GNNImpute uses graph attention convolution to aggregate multi-level similar cell information and implements convolution operations on non-Euclidean space on scRNA-seq data. Distinct from current imputation tools, GNNImpute can accurately and effectively impute the dropout and reduce dropout noise. We use mean square error (MSE), mean absolute error (MAE), Pearson correlation coefficient (PCC) and Cosine similarity (CS) to measure the performance of different methods with GNNImpute. We analyze four real datasets, and our results show that the GNNImpute achieves 3.0130 MSE, 0.6781 MAE, 0.9073 PCC and 0.9134 CS. Furthermore, we use Adjusted rand index (ARI) and Normalized mutual information (NMI) to measure the clustering effect. The GNNImpute achieves 0.8199 (ARI) and 0.8368 (NMI), respectively.

**Conclusions:**

In this investigation, we propose a single-cell dropout imputation method (GNNImpute), which effectively utilizes shared information for imputing the dropout of scRNA-seq data. We test it with different real datasets and evaluate its effectiveness in MSE, MAE, PCC and CS. The results show that graph attention convolution and autoencoder structure have great potential in single-cell dropout imputation.

## Background

With the development of single-cell RNA sequencing (scRNA-seq) technology, it provides an easy way to process tens of thousands of single cells in parallel while providing gene expression data with single-cell-level resolution [1–3]. The traditional RNA-seq technology cannot address complex tissues or organs at the cellular level because it measures the average expression of thousands of cells at the same time. Different from the traditional RNA-seq technology, scRNA-seq is widely used to study cell analysis, including cell heterogeneity [4], cell subgroups clustering [5, 6] and cell development trajectories [7]. Meanwhile, scRNA-seq technology can enhance the clinical diagnosis of the patient’s disease, and help doctors further customize treatment plans [5, 6, 8].

The scRNA-seq technology can produce single-cell-level resolution data. As a result of defects such as low capture rate and low sequencing depth, the sequencing library data contains a lot of noise [9, 10].

Compared with the next-generation sequencing data, scRNA-seq data usually contains a lot of zero expressions. These zero expressions can arise in two ways: One is that the genes are not expressed in the corresponding cells and the other is that some genes with low expression cannot be detected due to technical limitations. These events are called dropout events [11]. There are some reasons for dropout, including nonlinear amplification of mRNA, transcription efficiency when reverse transcription of mRNA to cDNA and low sequencing read depth [11–13].

In the downstream analysis of scRNA-seq data, the dimensional reduction and unsupervised clustering are always be used to infer cell development trajectories and identify rare cell clusters [14, 15]. However, dropout events will seriously affect the calculation of the distance between expression profiles, which leads to downstream results [16].

Recently, many methods have been developed to impute dropout in scRNA-seq data. For example, MAGIC [17] is based on Markov affinity-based graph, which uses similar cells and genes information to impute missing values. However, this method lacks robustness and cannot adapt to the nonlinear relationship with genes. Furthermore, DCA [18] is a neural network-based method that uses deep autoencoding networks for unsupervised learning. It performs zero-inflated negative binomial modeling on scRNA-seq data to solve the problem of noise and gene distribution. In addition, there are some imputation methods based on deep learning or statistical methods such as scVI [19], scImpute [20], and SAVER [21]. These methods can only apply to Euclidean space by using Euclidean spatial data, such as the expression matrix. But they cannot directly deal with Non-Euclidean spatial data like cell graphs [22–25].

Therefore, we propose a novel structure neural network named GNNImpute, which is an autoencoder structure network that uses graph attention convolution. By building a graph from the scRNA-seq data, GNNImpute uses graph attention convolutional layer to make a targeted selection of similar neighboring nodes. Then, it aggregates these similar neighboring nodes. The nodes in the graph can continuously transmit messages along the edge direction until stability is reached. In this way, GNNImpute enables the expression of the cells in the same tissue area to be embedded in low-dimensional vectors through the autoencoder structure. GNNImpute can not only capture the co-expression patterns between similar cells but also remove sequencing technical noise from imputing dropout, which improves the downstream analysis of scRNA-seq data.

## Methods

### The high-level approach

In scRNA-seq data, each cell has its own expression profile, and the expression profile of each cell is different and unique. But cells from the same tissue or with the same function usually have similar features. Therefore, when a dropout event occurs in any cell, it can be recovered by the gene expression profile of similar cells.

GNNImpute is a deep learning method based on a graph attention neural network. Different from MAGIC, GNNImpute introduces the attention mechanism that can assign weights to different similar cells according to attention coefficients. It can establish nonlinear relationships between genes by learning low-dimensional embedding of expressions through the autoencoder structure network. Compared with DCA, GNNImpute can learn the gene co-expression patterns of similar cells by aggregating information from multi-level neighbors. The co-expression patterns can help recover low-expressed genes. GNNImpute reduces the dropout noise and improves the gene expression profile of cells.

**Fig. 1:**
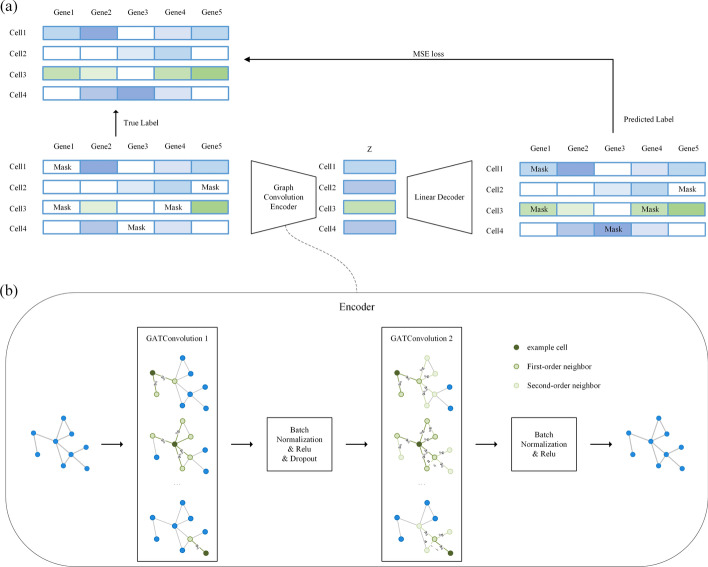
The structure of GNNImpute. **a** Shows the overall framework of the GNNImpute uses the network structure of the encoder and decoder. **b** Shows the encoder composed of two layers of graph attention convolutional layers

The overall structure of GNNImpute is shown in Fig. 1a. It is composed of an encoder and a decoder. Figure 1b shows that the encoder of GNNImpute has two graph attention convolutional layers, which are used to transmit the information of neighbor nodes. And the decoder consists of two linear layers. GNNImpute uses the masked expression matrix as the model input. The output of the model is used to calculate the loss value. And the parameters of the model are optimized by this value.

### Data preprocessing

GNNImpute uses the expression matrix of scRNA-seq data as input. As shown in Table 1, the expression matrix is an $nCells*nGenes$ scale. The rows represent different cells number, and the columns represent different gene sites. Each value in the matrix indicates the expression intensity of a gene in a cell. Due to the sparseness of scRNA-seq data, the expression matrix contains a very large number of zeros. When the expression values in row or column are all zero, it means that those cells or genes are no expression at all. We filtered these no expression values from the matrix, because these values may cause impurity interference and invalid information. Similarly, we filtered cells with overexpression in the matrix, which may be caused by incorrect counting or cell rupture after death.

**Fig. 2:**
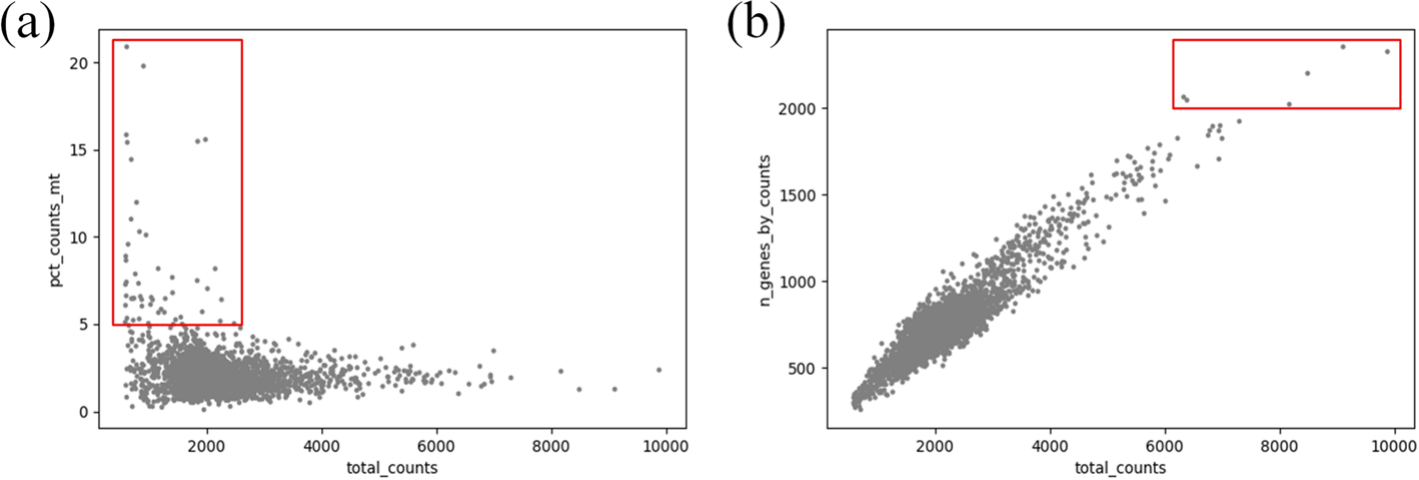
**a** shows the mitochondrial gene counts and total expression counts of the PBMC dataset. **b** shows the gene counts and total expression counts of the PBMC dataset. And the red boxs indicate outliers

In the data preprocessing, we use SCANPY [26] to filter the original matrix. We address the data in four steps. In the first step, cells with expression values less than 200 and genes with expression values less than 3 are filtered. Second, we filter the cells with overexpression of mitochondrial genes [27], as shown in Fig. 2a. Third, the cells with a high total expression count in the Fig. 2b should also be filtered. Finally, we normalize the filtered expression matrix, the purpose is to make each row (cell) in the expression matrix have the same value of expressions, and this value is the median of the values of expressions of all cells before normalization.

**Table 1:** Expression matrix of PBMC dataset (intercepted)

	MCL1	ENSA	GOLPH3L	HORMAD1	CTSS	CTSK	ARNT	SETDB1	CERS2	RP11-316M1.12	RP11-316M1.3	ANXA9	FAM63A	PRUNE	BNIPL
AAACATTGCACTAG-1	0	2	0	0	1	0	0	0	0	0	0	0	0	0	0
AAACATTGGCTAAC-1	1	0	0	0	2	0	0	0	0	0	0	0	0	0	0

### Build connection graph

**Fig. 3:**
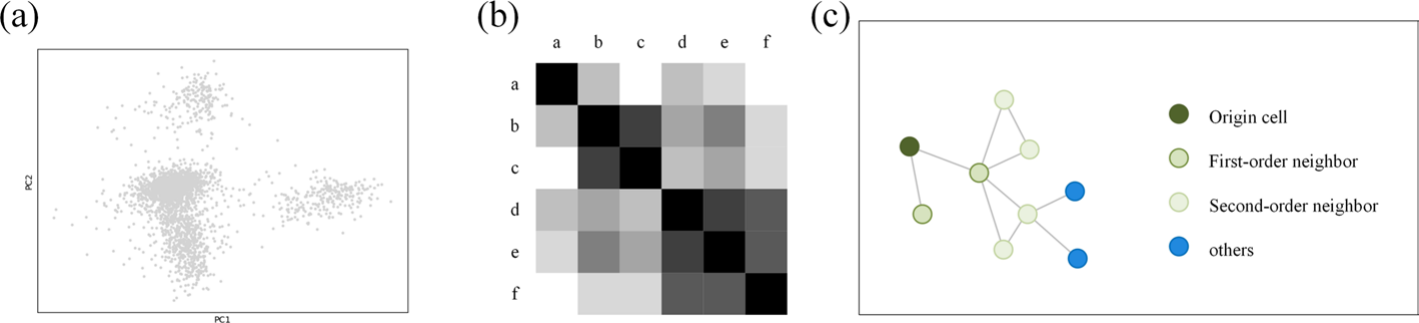
There are three steps to construct a connection graph. First, **a** shows the result of visualization after dimensionality reduction by PCA. Second, **b** is the distance matrix represented by a heat map. Third, **c** represents the K-nearest neighbor graph after selecting *K* neighbors

GNNImpute is a dropout imputation method based on a graph attention network. It is used to obtain gene expression from similar cells to recover the dropout event. In order to aggregate the cells with similar expressions, it is necessary to define a connection graph between the cells. In this graph, we use nodes to represent cells and we use edges to represent the similarity between cells. In this graph, cells can transmit information to adjacent cells. As shown in Fig. 3, the construction of such a graph is divided into three steps. The first step is to reduce the dimensionality of the expression matrix. Figure 3a shows the result of scRNA-seq data dimensionality reduction by Principal Component Analysis (PCA). After PCA, we can see that the cells are clustered according to similar expressions (Fig. 3a). We select the first 50 principal components as the GNNImpute input. The second step is to calculate the Euclidean distance between every two cells in the expression matrix. As shown in Fig. 3b, we get a heat map of $nCells*nCells$ scale. Heat map rows and columns represent different cells and the heat map color represent different cell distance. The color is deeper, the distance is closer. In the third step, we select *K* closest cells to construct graph edges. The *K* edges display a similar relation of cells. Through the above steps, we construct a cell-to-cell connection graph (K-nearest neighbor graph). We set the $K=5$ (The K number can be customized).

After constructing the graph, all graph cells have *K* cells with the most similar expression. We call these *K* cells the “first-level” neighbors. These K cells are adjacent to the origin cells. Similarly, there are also *K* cells in the “first-level” neighbors. We named the “second-level” cells. It doesn’t exist edges between origin cells and “second-level” cells, but the transferability of intermediate nodes can still indicate similarity. Figure 3c shows the origin cell and its neighbors. We use a two-layer graph convolution structure to transfer information within the range of “second-level” neighbors. This structure can not only maximize the aggregation of similar node information but also avoid over smooth node features.

### Multi-head graph attention convolutional layer

In order to aggregate the information of cells, we need a connection graph and graph attention convolutional layers. The essence of the graph convolutional layer is not to aggregate information around the original nodes, but aggregate nodes connected by edges. The calculation process of graph convolutional layers is as follows:

1 $$\begin{aligned} H^{(k+1)} = f(H^{(k)},A) = \sigma ({\hat{A}} H^{(k)} W^{(k)}) \end{aligned}$$

where *k* is the number of layers of graph convolution. *W* is the trainable weight. ${\hat{A}} = {\tilde{D}}^{-\frac{1}{2}} {\tilde{A}} {\tilde{D}}^{-\frac{1}{2}}$, Where ${\tilde{A}}$ and ${\tilde{D}}$ are the adjacency matrix and degree matrix of the cell-to-cell connection graph, respectively. The adjacency matrix ${\tilde{A}} = A + I$, *I* is the identity matrix, which means add self-connections to the adjacency matrix. The degree matrix ${\tilde{D}} _{ii} = {\textstyle \sum _{j}^{}} {\tilde{A}}_{ij}$. $\sigma$ is the activation function. ReLU is used as the activation function. $H^{(k)}$ is the input matrix of the k-th graph convolutional layer. When $k = 0$, $H^{(k)} = X$. In the GNNImpute, it is the expression matrix of scRNA-seq data.

Through the superposition of multiple graph convolutional layers, the information aggregation of multi-order neighbors can be achieved. We use two-layer graph convolution in the encoder, and the output of the encoder is as follows:

2 $$\begin{aligned} H^{(2)} = f(f(X,A),A)=ReLU({\hat{A}}ReLU({\hat{A}} XW^{(0)})W^{(1)}). \end{aligned}$$

To aggregate the information of neighbors more efficiently, we propose an attention model for neighbor nodes. By adding attention to neighbor nodes in the form of weights, this attention model achieves targeted aggregation of neighbor nodes. Specifically, the more similar the neighbor node is to the target node, the greater the attention coefficient obtained by the neighbor node. In this way, different weights are applied to different neighbors. The calculation of the attention coefficient is as follows:

3 $$\begin{aligned} e_{ij} = a(\overrightarrow{h_i} ,\overrightarrow{h_j}) = W\overrightarrow{h_i} \cdot W\overrightarrow{h_j} \end{aligned}$$

where $\overrightarrow{h_i}$ and $\overrightarrow{h_j}$ represent the features of node *i* and node *j*, which is the gene expression profile of the cells. And $e_{ij}$ represents the attention coefficient of cell *j* to cell *i*. *a*() is the attention calculation formula. It is used to calculate the similarity of every two nodes. we use the dot product as the attention calculation formula to calculate the similarity. *W* is a shared weight matrix. It transforms the input features into more advanced features, so that each node can obtain sufficient expressive ability. We only calculate the attention coefficient of cell *i* and cell *j*, where $j \in N_i$, and $N_i$ is the first-order neighbors of cell *i* in the cell-to-cell connection graph. Further calculation of multiple independent attention coefficients can be extended to the multi-head attention mechanism. In this attention mechanism, the attention coefficients are combined by calculate the average values to stabilize the learning process. The formula is as follows:

4 $$\begin{aligned} {\overrightarrow{h_i}}' = \sigma \left( \frac{1}{K} \sum _{K=1}^{K} \sum _{K \in N_i}^{} a_{ij}^kW^k\overrightarrow{h_i}\right) . \end{aligned}$$

In order to compare attention coefficients between different nodes, it is necessary to add the softmax function to standardize it:

5 $$\begin{aligned} a_{ij} = softmax_j(e_{ij}) = \frac{exp(e_{ij})}{{\textstyle \sum _{k \in N_i}^{}}exp(e_{ij})}. \end{aligned}$$

Combining the above (1), (2), (3), (4), (5) formula, the final attention weight displays as follows:

6 $$\begin{aligned} a_{(i,j)} = \frac{exp(LeakyReLU(a(\overrightarrow{h_i},\overrightarrow{h_j})))}{ {\textstyle \sum _{k \in N_i}^{}exp(LeakyReLU(a(\overrightarrow{h_i},\overrightarrow{h_j})))}}. \end{aligned}$$

### Architecture and training

**Fig. 4:**
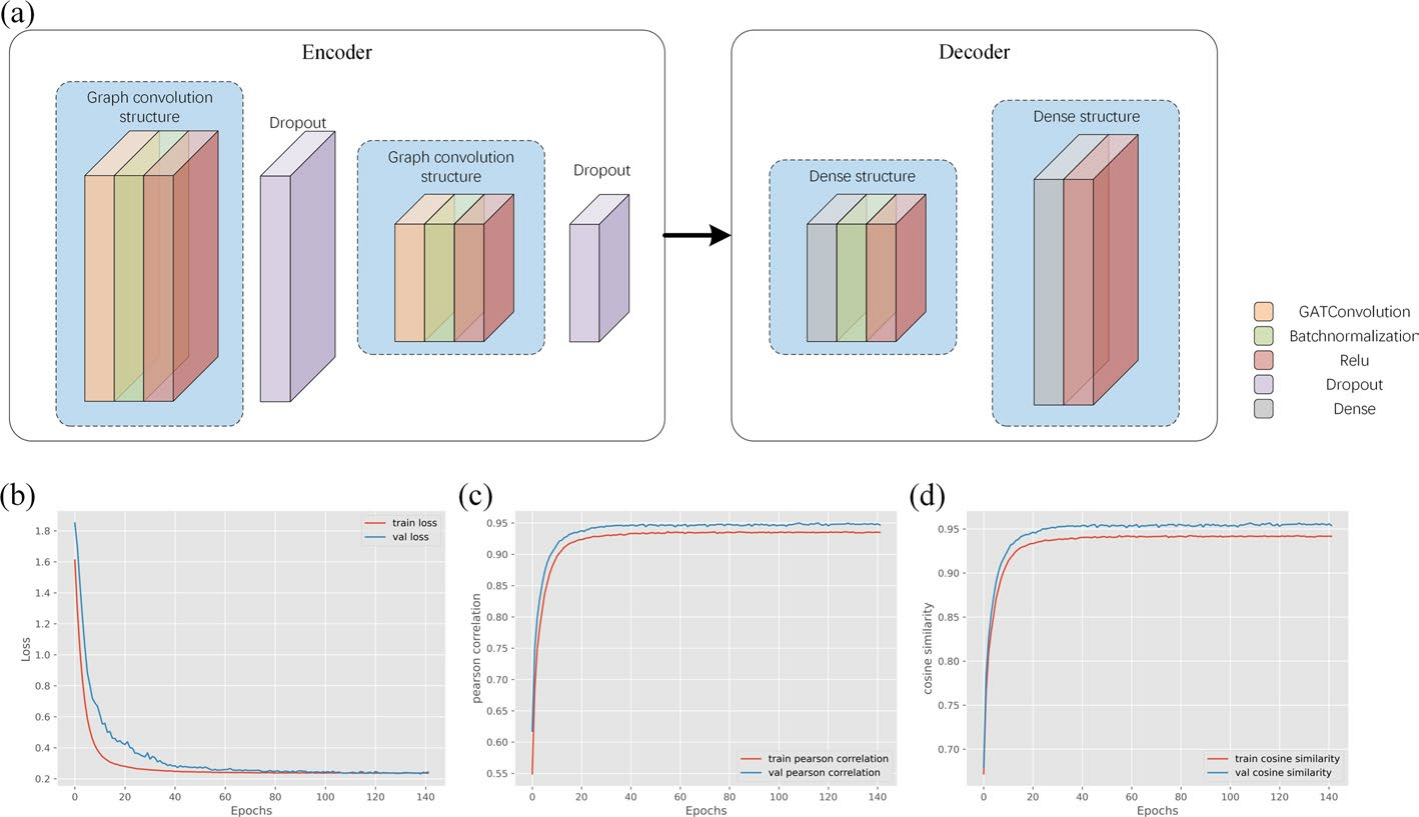
**a** is the model structure of GNNImpute. **b** is the loss curve of GNNImpute training and validation. **c** is the PCC curve of GNNImpute training and validation. And **d** is the CS curve of GNNImpute training and validation

GNNImpute builds the model with an autoencoder (encoder and decoder). The input layer and output layer of the model have the same number of nodes. In the hidden layer, the nodes are much lower than encoder and decoder nodes. Different from the traditional autoencoder structure, we make an improvement on the GNNImpute encode layer. We use graph attention networks in the GNNImpute encode layer instead of linear networks. As shown in Fig. 4a, there is the autoencoder structure used by GNNImpute. In the encoder, the input size of the first layer is the number of gene features of the cell, and the first layer output is 512. The second input size is equal to the first layer output (512). GNNImpute decoder is composed of three parts: linear layer, batch normalization layer and ReLU.

Further, GNNImpute adds dropout layers to combat the over-fitting problem of the model. GNNImpute introduces a multi-head attention mechanism to achieve targeted selection when aggregating neighbor nodes. This multi-head attention mechanism can stabilize the learning process and provide robustness for the model. It is noted that GNNImpute uses a semi-supervised learning method to recover from dropout events. The advantage of semi-supervised learning is that some labeled cells can provide soft labels for many other unlabeled cells, and it can help the model recover from dropout events more accurately.

The model can learn the potential features by minimizing the error between the reconstructed expression matrix and the original expression matrix. Meanwhile, the hidden layer can capture the distribution of the matrix and ignore invalid changes in the low-dimensional environment.

Because the dropout event is random, there are few dropout benchmarks. Therefore, we used a fair measurement method [28, 29]. This is a method of constructing a dropout benchmark by randomly masking the expression matrix. Using this fair measurement method can make various methods calculate the corresponding metrics. First, we process the expression matrix of the real scRNA-seq data to obtain the filtered matrix as the ground truth. Then, we randomly masked non-zeros based on a predetermined dropout rate. After two of the above steps, we can obtain the masked expression matrix and unmasked expression matrix. We can use the matrix data to train the GNNImpute model and validate the imputation effectiveness.

In the model training phase, we divide the PBMC dataset according to the ratio of 6:2:2. There are 1706 cells in the training set, 569 cells in the validation set, and 568 cells in the test set. Training set used to train the model, the validation set would be used to test the trained model, and the test set would evaluate the final model. The total parameters of GNNImpute will be adjusted according to the size of the dataset. When using the PBMC dataset, the total parameters of the model are 26.75 M. The loss function of the model is set to the mean square error loss function. The optimizer is Adam, and the learning rate is 0.0001. The maximum number of iterations is set to 3000. If the loss value of the validation set does not decrease in 200 consecutive iterations, it will interrupt training early. The training processes of the model are shown in Fig. 4b–d.

### Evaluation metrics

In the experiment, we use four metrics to measure the imputation ability of GNNImpute with the other four methods. The four metrics are mean square error (MSE), mean absolute error (MSE), Pearson correlation coefficient (PCC) and Cosine similarity (CS), respectively. MSE and MAE are used to show whether the imputed gene expression values are the same as the labels. PCC and CS are used to measure whether the express trend of the imputed matrix is consistent with the raw matrix. In downstream data analysis, we employ the Adjusted rand index (ARI) and Normalized mutual information (NMI) to measure the clustering results.

MSE:

7 $$\begin{aligned} MSE = \frac{1}{N}\sum _{i=1}^{N}(x_i-y_i)^2 \end{aligned}$$

where $x_i$ represents the imputed gene expression value, and $y_i$ represents the real gene expression value.

MAE:

8 $$\begin{aligned} MAE = \frac{1}{N}\sum _{i=1}^{N}\left| x_i-y_i \right| \end{aligned}$$

where $x_i$ represents the imputed gene expression value, and $y_i$ represents the real gene expression value.

PCC:

9 $$\begin{aligned} r = \frac{{\textstyle \sum _{i=1}^{N}}(x_i - {\overline{x}})(y_i - {\overline{y}})}{\sqrt{ {\textstyle \sum _{i=1}^{N}(x-{\overline{x}})^2}{\textstyle \sum _{i=1}^{N}(y-{\overline{y}})^2} } } \end{aligned}$$

where $x_i$ represents the imputed gene expression value, ${\overline{x}}$ represents the average gene expression after imputation, $y_i$ represents the real gene expression value, and ${\overline{y}}$ represents the real average gene expression.

CS:

10 $$\begin{aligned} cos(\theta ) = \frac{A\cdot B}{\left\| A \right\| \left\| B \right\| } = \frac{ {\textstyle \sum _{i=1}^{n}} A_i \times B_i}{\sqrt{ {\textstyle \sum _{i=1}^{n}(A_i)^2}} \times \sqrt{ {\textstyle \sum _{i=1}^{n}}(B_i)^2 }} \end{aligned}$$

where *A* and *B* represent the gene expression profile of the cell after imputation and the real gene expression profile of the cell respectively. And they are represented in the form of vectors. $A_i$ represents the expression value of the ith gene of the cell after imputation, and $B_i$ represents the real expression value of the ith gene of the cell.

### Datasets

We use four different real datasets in experiments. The real datasets list as following:

1. Human Frozen Peripheral Blood Mononuclear Cells (PBMCs), which from 10X GENOMICS. It contains 2900 cells and 32738 genes.
2. Mouse Brain cells published by Campbell (GSE93374). It uses Drop-seq technology to perform single-cell analysis on brain cells of adult mice, which contains 21,086 cells and 26,774 genes.
3. Mouse Brain cells published by Chen (GSE87544). It is the diversity analysis of mouse hypothalamic cells, which contains 14,437 cells and 23,284 genes.
4. Mouse embryo cell analysis published by Klein (GSE65525). It contains 2717 cells and 24,021 genes.

## Result

In order to validate the dropout imputation performance of different methods, we compare GNNImpute results with other five methods, including DCA, MAGIC, scVI, scImpute and SAVER. DCA uses a zero-inflated negative binomial distribution model to denoise the autoencoder network. This denoising network can solve the problem of count distribution, over-dispersion and sparsity. MAGIC is an imputation method based on Markov affinity-based graph, which imputes dropout values by sharing information among similar cells. scVI can capture the basic low-dimensional structure in the scRNA-seq data by introducing a robust latent variable model, which can eliminate the noise in the data. scImpute is a statistical method that can automatically identify possible dropout events and recover them. It can also exclude outliers without introducing new bias. SAVER uses regularized regression prediction and empirical Bayesian methods to recover the gene expression profile in noisy and sparse data. We conduct experiments on four single-cell sequencing datasets of humans and mice. To illustrate the imputation performance of methods, we use dropout recovery index, clustering, robustness to evaluate the results.

### Imputation evaluation

**Fig. 5:**
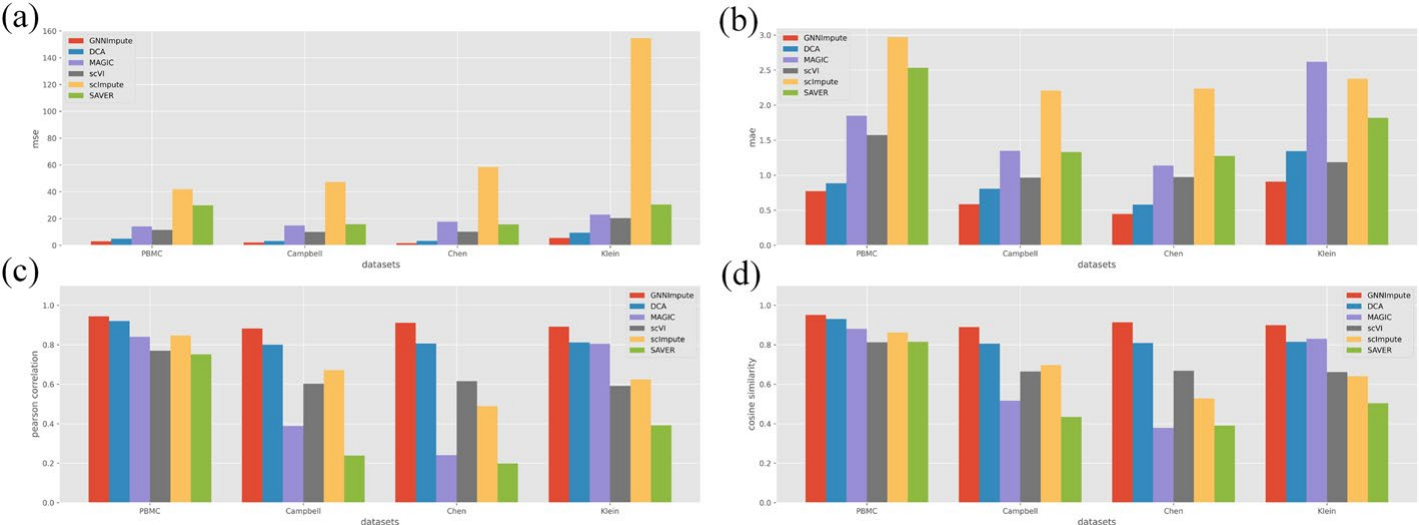
**a** shows the MSE between the gene expression value after imputation and the real gene expression value. **b** shows the MAE between the gene expression value after imputation and the real gene expression value. **c** represents the PCC between the gene expression value after imputation and the real gene expression value. **d** represents the CS between the gene expression value after imputation and the real gene expression value

By randomly masking the expression matrix on the four real datasets, we can obtain positive and negative training data. We compare the imputation performance of GNNImpute with the other five imputation methods using four real datasets. Figure 5 shows the performance of GNNImpute with the other five methods.

Overall, In the Fig. 5a, b, we can see the average MSE and MAE of GNNImpute can achieve 3.0130 and 0.6781. The results are better than DCA (3.0130 vs. 5.1888, and 0.6781 vs. 0.9036). The reason is that GNNImpute uses semi-supervised learning, which can learn from the labeled data to recover the dropout event. Since scVI is also a neural network method based on autoencoders, its performance is second only to DCA. For the scImpute, the MSE and MAE in the four datasets are the worst, because scImpute has an overall bias in the imputation. As shown in Fig. 5c, d, there are PCC and CS of GNNImpute and the other five methods in the four datasets. In PCC and CS, GNNImpute reaches the best result of 0.9073 and 0.9134 among all six methods. The performance is 8.69% and 8.71% better than the second place DCA (0.9073 vs. 0.8347, 0.9134 vs. 0.8402). It is because GNNImpute uses graph attention convolutional layer to aggregate information of similar cells. The performance of MAGIC, scImpute and SAVER in the four datasets are not stable. The average PCC and CS of MAGIC and SAVER on small datasets (PBMC, Klein) are 0.8226 and 0.5715 respectively. However, there are only 0.3146 and 0.2188 on the larger dataset (Chen, Campbell), which indicates that they cannot perform effective imputation on the large dataset. Another reason why scImpute may have an overall bias in imputation is that it has the worst performance in MSE and MAE but the PCC and CS are better than MAGIC and SAVER.

### Heat map and clustering evaluation

The purpose of imputation is to improve the downstream analysis of scRNA-seq data. Therefore, we use clustering results to evaluate the downstream analysis. We use two metrics (ARI, NMI) to measure the performance of cell clustering after imputation.

**Fig. 6:**
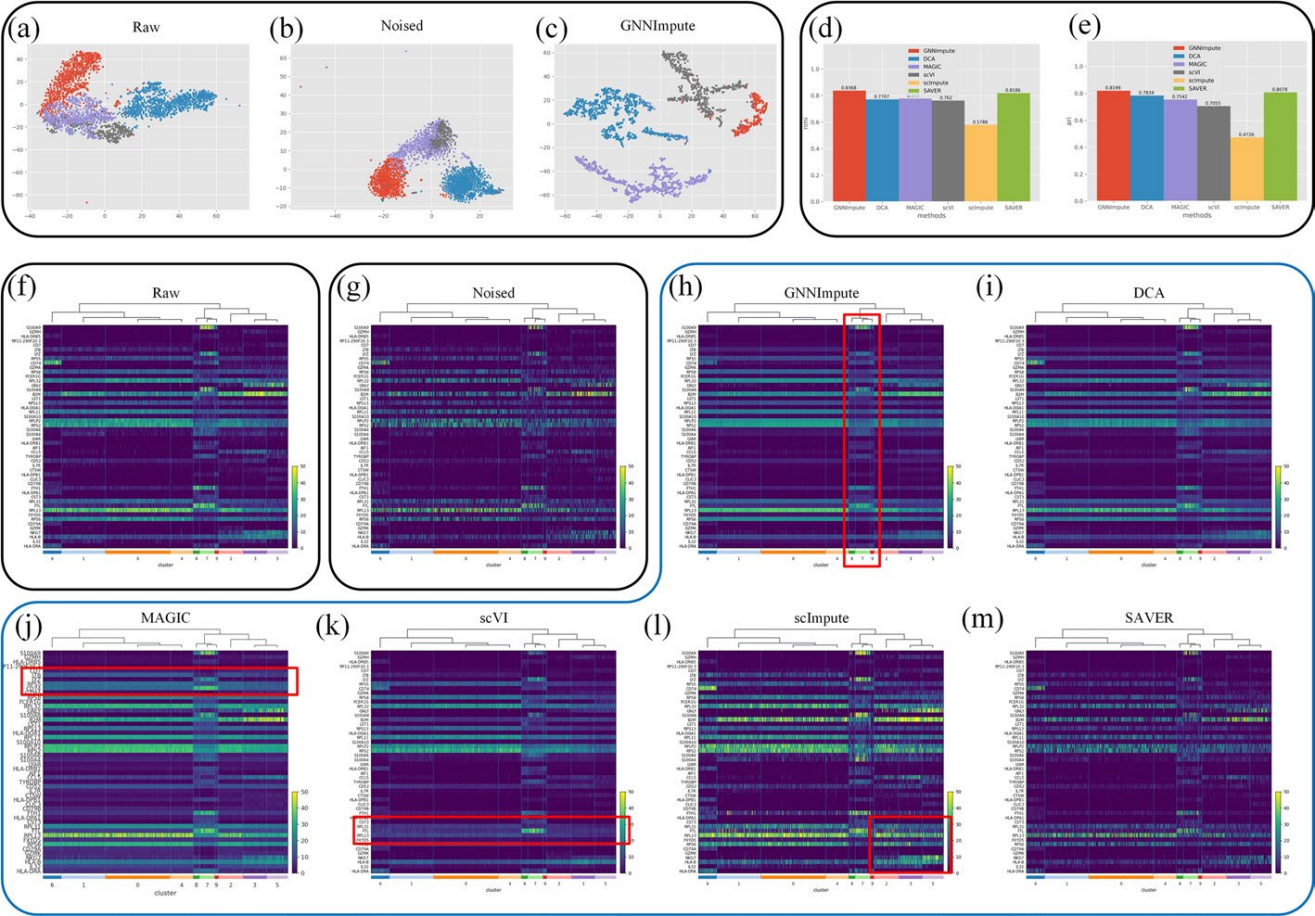
**a**–**c** show the visualizations of the raw matrix, noised matrix and denoised matrix after GNNImpute. **d**, **e** show the ARI and NMI of different methods. **f**, **g** show the heat maps of the raw expression matrix and noised matrix. **h**– **m** show the heat maps of the expression matrix imputed by GNNImpute and the other five methods

In the clustering analysis, we used the data published by Klein. They analyzed mouse embryonic stem cells, revealing in detail the population structure and the heterogeneous onset of differentiation after leukemia inhibitory factor (LIF) withdrawal. The cluster labels are determined by the intervals of LIF withdrawal (0, 2, 4, 7 days). The t-distributed stochastic neighbor embedding (t-SNE) algorithm is used to reduce the dimension of the expression matrix. And it can realize the visual analysis of clustering. In Fig. 6a–c, we show the figures of the raw matrix, noised matrix and the denoised matrix after GNNImpute imputation. The dimensions of these matrices are all reduced by t-SNE for visualization. The visual analysis of the noised expression matrix in Fig. 6b shows that four cell clusters have different degrees of mixing, and there is no obvious dividing line. But the expression matrix imputed by GNNImpute can separate different clusters, as shown in Fig. 6c. After imputing the matrices with different methods, we use k-means algorithm to measure the performance of matrix clustering. Then, we use ARI and NMI to measure the clustering results obtained by the k-means algorithm. GNNImpute reaches 0.8368 (ARI) and 0.8199 (NMI), which are at least 1.82% and 1.21% better than other methods (shown in Fig. 6d, e).

By calculating the gene heat map of all cells in the imputed expression matrix, the results can also be visualized to determine which methods can improve the downstream analysis of scRNA-seq data. Because the PBMC dataset does not have real cluster labels, we use Leiden algorithm to calculate pseudo labels. Then we find highly differentiated marker genes in each cluster by t-test based on the pseudo-labels. Finally, we select 50 marker genes most relevant to cluster classification in the PBMC dataset to measure the performance of GNNImpute and other five methods. Figure 6f, g shows the heat maps of the raw matrix and noised matrix. GNNImpute can recover the dropout events that occurred in different clusters, especially rare cell clusters (No. 7, No. 8 and No. 9 clusters, shown in Fig. 6h). The expression matrix after imputation by MAGIC shows that the large cell clusters are almost the same. The recovery of the dropout event is too smooth (such as LTB, RPS5 and CD74, shown in Fig. 6j). As a result, it lost the unique heterogeneity of scRNA-seq data. For scVI, it can only impute limited dropout values. The reason may be that low-expressed genes are mistaken for noise and ignored, such as RPL31 and RPS6 shown in Fig. 6k. scImpute can impute the dropout genes. But it changes the expression intensity of most genes, which further illustrates that scImpute imputes dropout values with a certain overall bias. The genes marked in Fig. 6l show that the expression intensity of these genes has been changed. SAVER does not perform obvious imputation. It may be that it cannot handle data with a high dropout rate.

### Robustness analysis under different dropout rates

Next, we evaluate the ability of the imputation method for scRNA-seq data under different dropout rates. The dropout rates are 10%, 20%, 30%, 40%, 50%, and 60%, using PBMC dataset with random mask expression matrix.

**Fig. 7:**
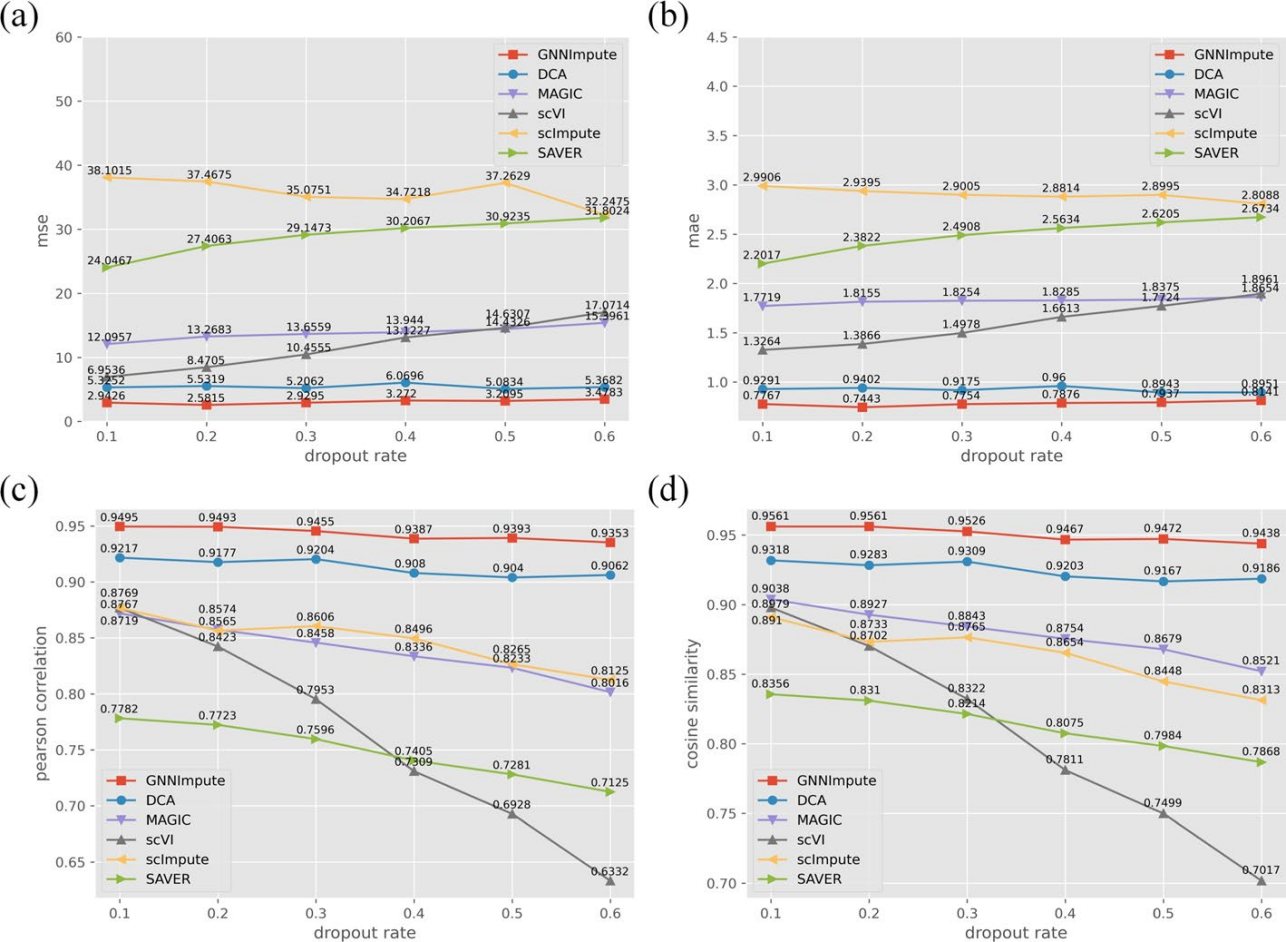
**a** shows the MSE of six methods at different dropout rates. **b** shows the MAE of six methods at different dropout rates. **c** shows the PCC of six methods at different dropout rates. **d** shows the CS of six methods at different dropout rates

Figure 7a–d shows the performance of the six scRNA-seq dropout imputation methods under different dropout rates. From Fig. 7 we can see that GNNImpute is not sensitive to the dropout rate. It can recover the most dropout events at a high dropout rate (60%). The MSE and MAE are 3.4783 and 0.8141. The PCC and CS are 0.9353 and 0.9438, respectively. After GNNImpute is DCA and MAGIC. Under different dropout rates, the MSE and MAE of DCA are hardly decrease. But the PCC and CS decrease by 1.7% and 1.4%. The MSE and MAE of MAGIC increased by 27.3% and 5.3%. And the PCC and CS decreased by 8.1% and 5.7%. The performance of scImpute and SAVER is in the middle. With the increase in the dropout rate, MSE and MAE show a clear increasing trend, while PCC and CS show a slowly decreasing trend. scVI is the most sensitive method for the dropout rate. MSE and MAE increased significantly (6.9536 to 17.0714 and 1.3264 to 1.8961). And both PCC and CS are decreased (0.8767 to 0.6332 and 0.8979 to 0.7017).

### Analysis of different training sets for semi-supervised learning

**Fig. 8:**
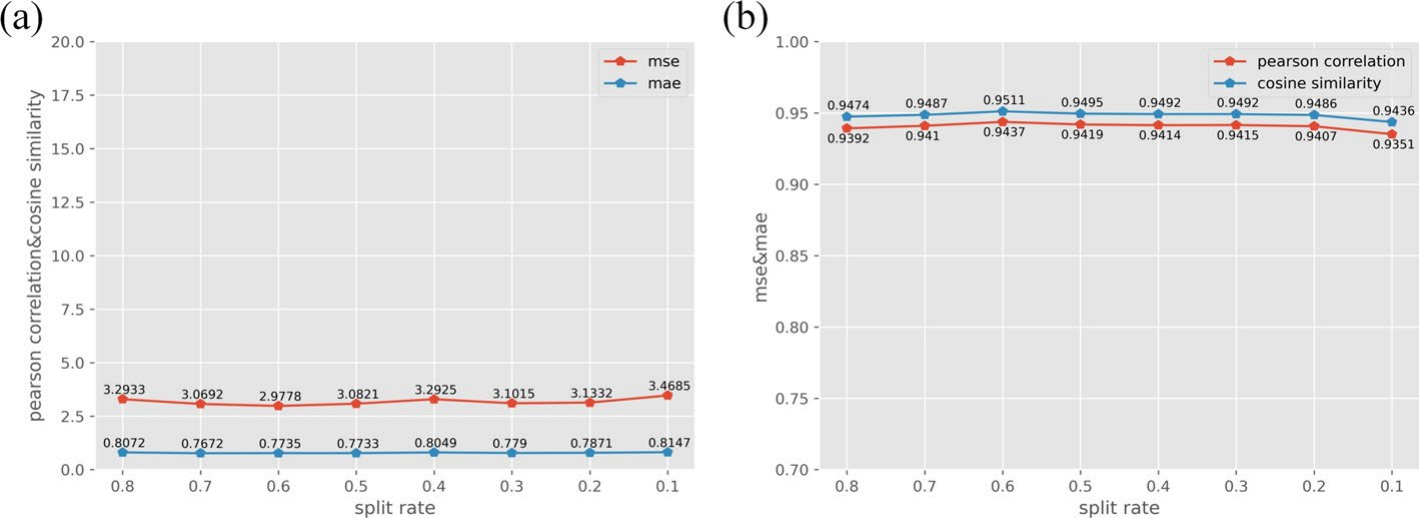
**a** shows the MSE and MAE of GNNImpute in different scales of training set. **b** shows the PCC and CS of GNNImpute in different scales of training set

GNNImpute uses a semi-supervised learning method to train the model and learn the dropout knowledge. The advantage of semi-supervised is that it can use only a small amount of labeled data and a large amount of unlabeled data for training, which greatly reduces the requirements for manually labeling data. In this experiment, we use 80 ~10% data with labels for model training. Even if only 10% labeled data is used, the model still shows great imputation performance, as shown in Fig. 8. The MSE and MAE are 3.4685 and 0.8147, and the PCC and CS are 0.9351 and 0.9436, respectively.

### Imputation performance in simulated data

To further evaluate the performance of GNNImpute, we evaluate the performance in simulated data. Following the previous work, we used the Splatter package [30] to generate two simulation datasets. The first dataset has 2 groups, and the second dataset has 6 groups. Both simulation datasets contain expression matrices of 4000 cells and 20,000 genes. Table 2 shows the MSE, MAE, PCC and CS of GNNImpute and the other five methods in simulated dataset (2 groups). GNNImpute is better than other methods on MAE, PCC and CS. Only on MSE, our method is slightly inferior to DCA (21.2961 vs. 19.9282), but compared with the other four remaining methods, our method still has a significant improvement. When we evaluate in the simulated dataset (6 groups), we can get similar results, as shown in Table 3. The reason for this phenomenon may be that the simulated data generated by the Splatter package is quite different from the real data. After calculation, we can confirm that the sparsity of the simulated data is much lower than the real data (simulated data: 0.47, PBMC: 0.94, Campbell: 0.89, Chen: 0.92, Klein: 0.66).

**Table 2:** Imputation performance in simulated data (2 groups)

	MSE	MAE	PCC	CS
GNNImpute	21.2961	**1.8432**	**0.9259**	**0.9344**
DCA	**19.9282**	1.8981	0.9092	0.9135
MAGIC	42.1510	4.1618	0.9022	0.9068
scVI	149.9295	2.7659	0.6282	0.6465
scImpute	99.0206	3.1611	0.7140	0.7088
SAVER	102.1126	3.3974	0.7962	0.8152

The bold values indicate the best or better scores that can be obtained through different methods under different indicators

**Table 3:** Imputation performance in simulated data (6 groups)

	MSE	MAE	PCC	CS
GNNImpute	25.7855	**1.9330**	**0.9202**	**0.9295**
DCA	**21.5326**	**1.9313**	0.9072	0.9113
MAGIC	45.0551	4.2573	0.8999	0.9050
scVI	147.4127	2.758	0.6272	0.6460
scImpute	99.4479	3.1622	0.7134	0.7085
SAVER	101.7722	3.3927	0.7951	0.8143

The bold values indicate the best or better scores that can be obtained through different methods under different indicators

### Analysis of attention mechanism of GNNImpute

In order to verify the effectiveness of the attention mechanism, we specially added experiments to evaluate our method and GCN architecture model (without attention). As shown in Tables 4 and 5, we evaluated the performance of five models on the PBMC dataset and Klein dataset. They are GNNImpute (GCN architecture without attention), GNNImpute (with 1 attention head), GNNImpute (with 3 attention heads), GNNImpute (with 5 attention heads) and GNNImpute (with 8 attention heads). The experiment method uses five independent repeated experiments to take the average value. The results of PBMC dataset in Table 4 show that the performance of the model using the attention mechanism is all better than the GCN model (without attention). In the evaluation of MSE and MAE, the model using the attention mechanism is at least better than GCN model (without attention) by 12.9% (3.3047 vs. 2.8800) and 3.6% (0.8022 vs. 0.7736). And they are also better than GCN (without attention) on PCC and CS (0.9436 vs. 0.9478, 0.9510 vs. 0.9547). Table 5 shows the improvement of the clustering effect of the attention mechanism on the Klein dataset. In the evaluation of ARI, the attention mechanism provided about 2% (0.7998 vs. 0.8155). For the evaluation of NMI, it is also better than GCN (without attention) by 1.6% (0.8204 vs. 0.8049). We also observed that the performance of GNNImpute (with 1 attention head) is not stable, so we recommend using the multi-head attention mechanism to stabilize the performance of the model.

**Table 4:** Imputation performance of GNNImpute and GCN architecture model (PBMC dataset)

	MSE	MAE	PCC	CS
GCN (without attention)	3.3047	0.8022	0.9436	0.9510
GNNImpute (1 attention head)	2.8800	0.7736	0.9478	0.9547
GNNImpute (3 attention heads)	**2.7767**	**0.7684**	**0.9494**	**0.9561**
GNNImpute (5 attention heads)	2.8748	0.7730	0.9480	0.9550
GNNImpute (8 attention heads)	2.8494	0.7699	0.9482	0.9551

The bold values indicate the best or better scores that can be obtained through different methods under different indicators

**Table 5:** Imputation performance of GNNImpute and GCN architecture model (Klein dataset)

	MSE	MAE	PCC	CS	ARI	NMI
GCN (without attention)	6.7994	0.8841	0.8805	0.8882	0.7998	0.8049
GNNImpute (1 attention head)	5.7850	0.9059	0.8779	0.8867	0.6945	0.7232
GNNImpute (3 attention heads)	6.1119	0.8725	0.8867	0.8942	0.8183	0.8204
GNNImpute (5 attention heads)	**4.9420**	0.8629	**0.8950**	**0.9024**	0.8155	0.8311
GNNImpute (8 attention heads)	6.4669	**0.8592**	0.8921	0.8988	**0.8202**	**0.8366**

The bold values indicate the best or better scores that can be obtained through different methods under different indicators

## Discussion

On the high level, imputing the dropout of scRNA-seq data is a process of denoising the expression matrix data of the raw scRNA-seq library. Many existing approaches show that deep learning methods, especially autoencoders, can effectively denoise data.

Our GNNImpute method extends this high-level approach to the case of Non-Euclidean spatial data like cell graphs. By reconstructing the expression matrix of scRNA-seq data, GNNImpute can establish a learning mechanism between the input and output of the model. It allows our model to capture non-linear relationships between genes and make better utilization of data.

Usually, the dropout imputation performance is affected by the sparseness of scRNA-seq data. For noise data, it is usually more difficult to impute dropout values. However, GNNImpute shows excellent performance compared with other methods. GNNImpute compensates for the lack of low expression intensity of some genes by aggregating the features information of similar cells. Meanwhile, it can recover the dropout events in the scRNA-seq data and remain the specificity between cells to avoid excessive smoothing of expression.

Compared with other dropout imputation methods, GNNImpute has great adaptability for addressing the different sizes of datasets (especially large datasets). The difficulty in processing large datasets is that there are many cell types, and each cell contains a lot of gene information. Since GNNImpute uses a neural network model, it can capture important features from all information, and then reduce the dimensionality to ignore unimportant features.

Moreover, GNNImpute is a semi-supervised learning model, which does not require manually labeled data. It can be trained with few labeled data. This model only uses 10% of the dataset for training can still achieve great results.

## Conclusions

In this paper, a novel imputation method based on graph attention convolution is proposed, which is a semi-supervised learning method using an autoencoding structure network. GNNImpute focuses on determining the similarity between cells and constructs a connection graph to capture the features of similar cells. This method also introduces an attention mechanism of weighted neighbor nodes to select the cell node with the most useful features information. In the experiments of four datasets, the performance of GNNImpute is better than other existing methods for four metrics of MSE, MAE, PCC and CS. When we explore the limits of GNNImpute, we find that it cannot provide the interpretability of cell clusters. Future investigations will focus on how to make GNNImpute more explainable.

## Data Availability

The datasets used in this study are publicly available. Single-cell library data and raw count expression matrices of PBMCs are downloaded from 10X GENOMICS (https://www.10xgenomics.com/resources/datasets/frozen-pbm-cs-donor-a-1-standard-1-1-0). The mouse brain cell data released by Campbell is available at Gene Expression Omnibus (GEO) under accession code GSE93374. The single-cell data and expression matrix data of mouse brain cells published by Chen are available in GEO under accession code GSE87544. The mouse embryo single-cell data published by Klein was downloaded from GEO, and the accession code is GSE65525. The source code in this paper is available at https://github.com/Lav-i/GNNImpute.

## Implementation

GNNImpute is implemented in Python 3 using deep learning framework PyTorch Geometric [30]. Training on CPU or GPU is supported using PyTorch and PyTorch Geometric.

## Simulated scRNA-seq data

We used the Splatter [31] package to generate simulation datasets. The parameters used in the generated two sets of simulation data are as follows. For two group simulation: nGroup = 2, dropout.mid = 5, dropout.shape = − 1, dropout.type = “experiment”, de.facScale = 0.25, nGenes = 20000, batchCells = 4000. For six group simulation: nGroup = 6, dropout.mid = 5, dropout.shape = − 1, dropout.type = “experiment”, de.facScale = 0.25, nGenes = 20000, batchCells = 4000.

## Abbreviations

scRNA-seq: Single-cell RNA sequencing
RNA-seq: RNA sequencing
PCA: Principal component analysis
RuLU: Rectified linear unit
MSE: Mean square error
MAE: Mean absolute error
ARI: Adjusted rand index
NMI: Normalized mutual information
t-SNE: t-distributed stochastic neighbor embedding
